# Insights from the development of an online toolkit to promote community-engaged substance use research in the Southwest Borderlands Region

**DOI:** 10.1017/cts.2026.10804

**Published:** 2026-07-16

**Authors:** Andrew Gorvetzian, Maria Sanchez, Christina Phillips, Joseph Allbright, Alexandra Yonkovich, Cynthia Killough, Randy Benally, Janet Page-Reeves, Mary Alice Scott, Kimberly Page, Ivan de la Rosa

**Affiliations:** 1 Anthropology, The University of New Mexico Department of Anthropology, Albuquerque, NM, USA; 2 Internal Medicine, The University of New Mexico Health Sciences Center, Albuquerque, NM, USA; 3 Social Work, New Mexico State University, Las Cruces, NM, USA; 4 Clinical and Translational Science Center, University of New Mexico Health Sciences Center, Albuquerque, NM, USA; 5 Southwest Center for Advancing Clinical and Translational Innovation, University of New Mexico, Albuquerque, NM, USA; 6 Family and Community Medicine, The University of New Mexico Health Sciences Center, Albuquerque, NM, USA; 7 Center for Health Services Research, University of North Carolina Wilmington, Wilmington, NC, USA; 8 School of Social Work, New Mexico State University, Las Cruces, NM, USA

**Keywords:** community engagement, substance use research, participant diversity in clinical trials, Borderlands region, interdisciplinary scholarship

## Abstract

**Background:**

Underrepresented communities in the Southwestern United States Borderlands region face disproportionate substance use disorder (SUD) burdens yet remain largely excluded from clinical trials. No existing resource integrates health beliefs, research literacy, and vulnerability frameworks to support diverse community engagement in SUD clinical trials.

**Methods:**

We developed the Exploring Health Beliefs for Community Engagement and Diversity in Clinical Trials (EXPLORE) Toolkit through a multi-phase process (2021–2024) including systematic review of existing toolkits, domain development, platform design, individual interviews (*n* = 10), and two rounds of theater testing with community health workers serving Hispanic communities in Southern New Mexico (*n* = 60) and American Indian communities in Northern New Mexico (*n* = 25). Pre/post surveys assessed feasibility, acceptability, community interest, and research literacy. Ripple Effects Mapping evaluated the development process.

**Results:**

Domain-specific toolkit importance ratings averaged 4.48–4.61 on a 5-point scale; 79% of participants rated the toolkit “very important” for promoting culturally competent research, and 64% were “very likely” to recommend it. Post-theater testing scores improved for perceived feasibility of conducting clinical research in participants’ communities (pre: 7.19 vs. post: 7.94, 10-point scale) and research terminology understanding (pre: 7.97 vs. post: 8.74). Qualitative themes included cultural appropriateness, the role of community gatekeepers, and confidentiality concerns in rural and border communities.

**Conclusions:**

The EXPLORE Toolkit demonstrates promise for bridging researcher–community gaps in SUD clinical trials. Findings highlight the value of interdisciplinary, community-centered development and the need for ongoing cultural adaptation. The toolkit is freely available at https://www.exploretoolkit.org/.

## Introduction

The Southwestern United States Borderlands region, encompassing southern New Mexico and adjacent areas, is characterized by high rurality, deeply rooted Hispanic and American Indian communities, and substantial migrant Hispanic/Latino populations. These communities face a devastating SUD crisis compounded by pervasive health disparities, creating urgent need for innovative, rigorously evaluated solutions [1–3]. Despite this burden, underrepresented populations remain largely excluded from clinical trials, limiting both the generalizability of research findings and the translation of evidence-based treatments into affected communities [4–9]. Documented patterns of exclusion are well-established: racial and ethnic minorities, including Hispanic/Latino and American Indian populations, have been consistently underenrolled relative to their disease burden across trial types; [4,5] rural populations face structural barriers including geographic distance from trial sites, limited research infrastructure, and historical mistrust that compound their exclusion [10]; and in SUD-specific research, individuals with polysubstance use are frequently excluded through restrictive eligibility criteria that prioritize single-substance diagnoses, reducing generalizability to real-world clinical populations [11–13].

Underrepresentation in clinical trials stems from multiple intersecting factors: limited knowledge about clinical research, mistrust of researchers, inadequate healthcare access, geographic constraints, and socioeconomic barriers including poverty and discrimination based on race, gender, and sexual orientation [4–6,8,10,14,15]. Although tools for increasing research engagement exist, no prior resource specifically aimed at advancing inclusion of diverse and minority populations in SUD clinical trials employed a conceptual framework integrating health beliefs, research literacy, and vulnerability.

To address this gap, an interdisciplinary team of epidemiologists, social workers, anthropologists, and public health professionals from the University of New Mexico (UNM) and New Mexico State University (NMSU) developed the EXPLORE Toolkit. The Toolkit employs a strengths-based approach to community engagement, addressing equity, stigma, and participation barriers while promoting culturally sensitive, community-centered research practices. It serves researchers, community health workers (CHWs), and other frontline workers seeking to engage underrepresented populations in clinical trials throughout the region and beyond.

### Interdisciplinary approaches to community-engaged research

The fields of public health, epidemiology, and clinical trials research demonstrate growing commitment to meaningful community engagement. Contemporary calls for interdisciplinary approaches to complex issues have fostered collaborations among researchers from many disciplines, including social work, psychology, and social and medical anthropology, offering promising pathways for improving inclusion of diverse populations in clinical research while enhancing the relevance and translation of research findings into practice [10,14,16,17].

Before describing toolkit development, it is important to clarify what is meant by “community.” Most commonly, definitions coalesce around a “group of people with diverse characteristics who are linked by social ties, share common perspectives, and engage in joint action in geographical locations or settings.” [18] The U.S. Centers for Disease Control and Prevention broadly defines it as a “geographic area, a group of people with shared interests, or a feeling of teamwork and fellowship.” [19] However, some researchers argue that focus should not be geographic, but on a common shared identity [20], and also on the concept that a single individual may belong simultaneously to many different communities – social, cultural, ethnic, vocational, spiritual, physical, intellectual, and economic [21]. In health research The U.S Centers for Disease Control and Prevention broadly defines it as a “geographic area, a group of people with shared interests, or a feeling of teamwork and fellowship.” [19] In health research equity contexts, “community” typically refers to non-academic partners, participants, and other interested parties, with emphasis on inclusivity. However, individuals belong to multiple, overlapping, fluid, and sometimes contested communities – a complexity often more pronounced within minoritized groups. Investigators should therefore engage collaborators early to establish a concrete, shared understanding of “community” relevant to their specific project, including parameters for data ownership and sharing, and the resulting framework should honor the outcomes of this collaborative process.

## Methods

### Toolkit design

Development began in September of 2021 and unfolded through seven sequential phases over three years: (1) review and evaluation of related toolkits and sources; (2) development of EXPLORE domains; (3) design of online toolkit and platform; (4) focus groups for beta version; (5) toolkit revisions; (6) theater testing; and (7) final revisions. This methodology reflects the insights of community-engaged scholars cited above and the toolkits we reviewed. However, we did not explicitly emulate any prior intervention or case study. The project began because there were no existing resources integrating health beliefs, research literacy, and vulnerability frameworks to support diverse community engagement in SUD clinical trials for the Southwestern US. The conversations and insights that our interdisciplinary team shared in weekly meetings over the course of three years shaped our methodology.

#### Phase 1: review of existing resources

The team conducted a narrative review of existing toolkits with keyword searches for toolkits on public health, community engagement, patient-centered outcomes, health beliefs, and strategies for engaging underrepresented communities in research. Seventeen tools, toolkits, and frameworks met search criteria. Each was systematically analyzed for mission and goals, components, engagement methods and principles, adaptable tools, user experience and design, and limitations (Table 1). Rather than duplicating existing work, the team aimed to create a comprehensive “one-stop shop” integrating previously developed tools with new contributions.

**Table 1.** Main Principles and components of existing toolkits Table 1 long description.

Name of toolkit	Mission/aim of toolkit	Methods/principles of engagement	Components of toolkit	Other useful info	Unique elements
ReachNet	The involvement of patients, caregivers, clinicians, and other healthcare stakeholders throughout the research process – from topic selection through design and conduct of research, to dissemination of results. Helping people make informed decisions to improve health care delivery and health outcomes.	Patients can contribute to and prioritize research topics; can participate beyond being subjects and more as consultants; be connected to the clinical research teams in meaningful ways	Script for medical assistant; Data use policy; Patient Consent forms	This is an upstream approach. Key is: who controls the narrative. How to include voices input for trials that are already designed.	Clinic champion (pg. 18): a role for how to integrate patients, providers, and other stakeholders
Brandeis Community Engagement Studio (CES) toolkit	Providing an outline for an interactive, consultative model for community involvement in which community experts are seen as collaborators in research design, not subjects of studies	Key members of CES team: Community navigator (navigates community relationships with academic group); science navigator (works with investigators to facilitate communication with stakeholders); manager (works with navigators to ensure effective implementation); Facilitator for Studio testing, Researcher presenting research, and community experts (pgs. 12–18 for more details)	Facilitator CES guide; Tools for Recruiting Researcher; Researcher CES guide; Tools for recruiting community experts; Community Expert CES guide; Consent forms; CES evaluation survey		Good table on page 7 distinguishing between focus group and CES
Gathering of Native Americans (GONA) toolkit	Complementing the guide above, this toolkit offers dozens of materials for team building, trust building, and engagement materials for facilitators working with Native American groups		Toolkits for Belonging, Mastery, Interdependence, Generosity and Healing from Intergenerational trauma; also one geared towards youth		Offers concrete examples of what centering “Indigenous ways of knowing” looks like in practice
Rain barrel communications participatory research toolkit^a^	A toolkit with dozens of specific tools for participatory action research, with outlines of the tools, reflections from experiences of using the tools, and other insights. Tools have been used for myriad health issues across the world, including female genital mutilation, nutrition, AIDS, malaria, etc.	They can be used to conduct participatory situation assessments, monitor the extent to which interventions are being implemented according to plan or to measure effectiveness, i.e., changes in behavioral and social outcomes. The strength of these tools lies in the fact that they can be integrated directly into individual and social change communication programming, providing participants the ability and skills to create and analyze data. Many of the tools are specifically designed to build teamwork and to make research an enjoyable exercise. Additionally, these tools can be used to examine societal and cultural factors which are difficult to understand and decipher using traditional methods. They allow both participants and researchers to see the world in a different light. (direct citation from intro to document)	The toolkit is a list of all of the tools used. Description of tool, rationale, application, and tips for use. See document for all of the tools		Too many tools to look at, could be useful for teams to go through and see if any are useful. This is a good example of how to lay out tools, how to apply, and how to use the tools and what to expect.

a This toolkit, which was developed in coordination with USAID, is no longer accessible online. From their website: As of March 15 2025, The Communication Initiative (The CI) platform is operating at a reduced level, with no new content being posted to the global website and registration/login functions disabled. (La Iniciativa de Comunicación, or CILA, will keep running.) While many interactive functions are no longer available, The CI platform remains open for public use, with all content accessible and searchable until the end of 2025. Please note that some links within our knowledge summaries may be broken due to changes in external websites. The denial of access to the USAID website has, for instance, left many links broken. We can only hope that these valuable resources will be made available again soon. In the meantime, our summaries may help you by gleaning key insights from those resources (https://global.comminit.com/content/participatory-research-toolkit).

#### Phase 2: domain development

The team met weekly to discuss the development of our toolkit. As we analyzed existing toolkits, we selected tools, themes, and layouts to incorporate into our design. These discussions yielded the insight that our toolkit should serve dual purposes: providing researchers with accessible tools for project design and execution and functioning as an educational resource about research processes and the broader socioeconomic, historical, and political factors shaping research engagement. In light of that conclusion, we organized our toolkit around the five domains presented in Table 2. Table 3 provides an example of the thematic areas we developed to identify structural forms of discrimination that affect participation, and Table 4 provides an example of how we built on those themes to develop a strengths-based approach to addressing those barriers.

**Table 2.** Domain names and descriptions Table 2 long description.

*Domain 1: preparing researchers to go into communities*	Introduces the toolkit’s purpose. Addresses three primary user groups: experienced researchers seeking greater participant diversity; clinicians and professionals involved in clinical trial recruitment or referral; and community health workers (CHWs) who interface directly with community members.
*Domain 2: structural factors that influence participation*	Presents five interconnected thematic areas – geography; racism, skepticism, and distrust of researchers; economic and material factors; education, stigma, and health literacy; and gender and sexuality – illustrating how social/economic policies, healthcare resource distribution, and social stratification create barriers to participation among marginalized populations.
*Domain 3: building on strengths to support participation*	Operates in parallel with Domain 2. Offers a framework for reframing perceived barriers as community assets and provides practical strategies for leveraging these strengths in clinical trial design and implementation.
*Domain 4: engagement and communication methods*	Features two illustrative vignettes depicting hypothetical researcher–community interactions, highlighting the critical role of language, context, and social dynamics in community engagement.
*Domain 5: tools and resources*	Provides comprehensive implementation resources: tables of tools organized by domain and theme, links to existing toolkits, original team-developed tools, and curated external resources supporting focus group facilitation, trust building, clinical trial education, and pre/post-trial evaluation.

**Table 3.** Themes and sub-themes explored in Domain 2: structural forms of discrimination Table 3 long description.

Theme	Subtheme	Example
Geography	Transportation instability	Having limited access to transportation could make it challenging for an individual to make it to their appointments for clinical trials if they are conducted in person
Racism, skepticism, and distrust of researchers	Skepticism of the medical system	Medical providers often operate under outdated approaches with stigmatizing beliefs about substance use and substance use treatment which cause patients to feel judged and avoid healthcare systems.
Economic/material factors	Poverty	Not having enough money to live comfortably could impact an individual’s decision to participate in clinical research due to time constraints and perceived value for giving their time based on the compensation.
Education, stigma, and health literacy	Health misinformation/ literacy	Misinformation has been a growing concern for the scientific community with the widespread use of social media and news platforms, which are pervasive and often provide incorrect information. This prevalence could limit the trust that an individual has in the motivation for the trial and potential outcomes.
Gender and sexuality	Gender Identity	Historically, female/woman-identifying individuals absorb much of the household labor and responsibility within a family unit, including childcare, which can limit the amount of time they are able to give to participate in clinical trials.

**Table 4.** Domain 3: building on a strengths-based to support participation Table 4 long description.

Barrier	In smaller communities it is more likely that people know each other or of each other. This can discourage participation in substance use research to avoid stigma and discrimination.
Strength	Video conferencing and other virtual communication resources have become more common in the wake of the COVID-19 pandemic. These tools facilitate more discrete participation in research.
Strategies	Virtual resources can reduce potential stigma against participating in clinical trial research as well as reduce barriers such as geographic distance from a research site. Virtual technology allows participants to be involved with privacy. Trials may be designed to reduce the amount of fact-to-face contact. There are a growing number of resources available for “Decentralized Clinical Trials” that may be well suited for rural areas. If your research takes place at a fixed site, consider how you can create a private entrance, or “backdoor” into the site to maximize privacy and confidentiality.

### Toolkit testing

#### Focus groups

Following initial Toolkit development, the team recruited potential users from Southern New Mexico, including healthcare clinics, organized community groups (e.g., the Doña Ana Wellness Institute), CHWs, and academic researchers. Recruitment of clinical researchers proved challenging given the limited clinical trials infrastructure in the region, and the ongoing COVID-19 pandemic further constrained in-person focus groups. The team therefore shifted to individual videoconference interviews (*n* = 10), ultimately including nursing professionals, frontline providers including Emergency Medical Technicians, and CHWs. These sessions yielded valuable insights regarding user experience and suggested the Toolkit’s utility could extend beyond its originally intended audience.

#### Theater testing

Following incorporation of the interview feedback, the team conducted theater testing to assess usability, content, and relevance. In health intervention design, theater testing evaluates the feasibility, acceptability, and potential effectiveness of an intervention or research approach before full implementation through metaphorical “staging” of the research process rather than theatrical performance [22,23]. The team identified CTN-107 (NCT05123027), a NIDA Clinical Trials Network study assessing a peer intervention linking opioid overdose survivors to treatment for opioid use disorder (OUD) [24], as a prototype study for Toolkit evaluation, given its potential feasibility in New Mexico.

The team partnered with the New Mexico Department of Health (NMDOH), Office of Community Health Workers (OCHW), focusing on Southern New Mexico. Community Health Workers (CHW) were selected for testing due to their integral role in New Mexican communities and their position as trusted facilitators and gatekeepers [25,26]. Building on previously established relationships, the Community Engagement Liaison Specialists at the Clinical and Translational Science Center facilitated connections with Promotoras de Salud (“*Promotoras*”; CHWs working with Hispanic/Latino communities),

Theater Test 1 included 60 promotoras recruited during a scheduled meeting in January 2024 in a “*colonia”* (rural unincorporated settlement) in Doña Ana County [27]. Written informed consent was waived by the UNM HSC IRB, as theater testing presented minimal risk and all data were collected anonymously. Information sheets in English and Spanish detailing procedures, risks, and benefits were provided to all participants. Two Spanish-speaking investigators (KP and IdlR) facilitated the session, conducted primarily in Spanish with simultaneous English translation for the small minority (<6) of non-Spanish speakers. Participants each received a $50 merchandise card.

Theater Test 2, conducted at the invitation of the NMDOH OCHW Director, included 25 CHWs serving American Indian communities in Northern New Mexico. This session was conducted in English with smaller group sizes to enable more detailed individual feedback. Participants used post-it notes to record thoughts before verbal group discussions, and the same pre/post evaluation tools were used.

#### Theater testing activities

Each session began with facilitators introducing the SW CTN EXPLORE project and its goals. Participants received bilingual Toolkit copies and electronic access via QR code. The team then staged a “request to consider research” intervention, presenting CTN-107’s study components including informed consent review and implementation procedures. Participants were randomly assigned to four breakout groups, each focusing on a specific Toolkit domain, and analyzed vignettes aligned with the intervention using their assigned domain’s tools. Groups then reconvened to share findings and synthesize feedback.

#### Measures

Pre- and post-evaluations assessed attitudes and perceptions using 10-point scales for feasibility, acceptability, community interest level, and research literacy (e.g. understanding of terms – randomization, informed consent, protocol, and institutional review board). Following toolkit testing, participants rated usability, cultural relevance, dialogue facilitation, informational value, and likelihood of recommending the toolkit on 5-point scales and rated the importance of each domain topics.

#### Ripple effects mapping

Ripple Effects Mapping (REM), a participatory evaluation technique was used to assess toolkit development process [28–30]. REM illuminates interconnections between program elements, activities, and impacts, creating visual “maps” that illustrate change pathways and capture unforeseen impacts that traditional evaluation methods may miss. Using XMind 8 Pro Mind Mapping Software, the team conducted a retrospective evaluation discussion in July 2023, guided by four questions: (1) What did we learn through toolkit creation? (2) What occurred during the development process? (3) How did this project impact participants? (4) How did this project influence our research approaches?

#### Analyses

Pre-/post-survey comparisons were analyzed using Wilcoxon signed-rank tests, appropriate for ordinal scale data with paired observations. Effect sizes were calculated as *r* = Z/√N. Analytic sample sizes varied by item (range: *n* = 54–64), reflecting participants with complete paired responses. Given high baseline scores on several items suggesting ceiling effects and the potential for social desirability bias in group administration settings, pre-/post-comparisons are interpreted descriptively rather than inferentially.

We conducted thematic analysis on the qualitative feedback from our two theater testing sessions with three study team members serving as coders. We used Microsoft Excel to identify and code themes and sub-themes from the feedback, organizing the results in a coding matrix that mapped participant responses against identified themes. The three coders demonstrated high agreement in their coding approach, with any discrepancies resolved through resolution by the lead coder.

## Results

### Quantitative results

Evaluation of the EXPLORE Toolkit revealed strong positive participant responses across multiple dimensions (Table 5). Usability received the highest rating (*M* = 4.26, SD = 0.89 on a 5-point scale), followed by ability to promote dialogue about SUDs (*M* = 4.10, SD = 1.18) and cultural relevance (*M* = 4.07, SD = 1.11). Sixty-four percent of participants were “very likely” to recommend the Toolkit to others, 15% were “likely” to recommend it, and 6% were unlikely to recommend it. All domain topics received strong endorsement, with mean importance ratings ranging from 4.48 (Domain 1) to 4.61 (Domain 3). A majority (79%) of participants found the Toolkit’s guidance valuable for researchers working with communities; the remaining 21% found it somewhat valuable. No participants rated it as lacking value.

**Table 5.** Toolkit Impressions and importance of toolkit domain topics Table 5 long description.

Impressions and views	Score	
	Mean	SD
Toolkit usability	4.26	0.89
Cultural relevance	4.07	1.11
Promotes dialogue	4.10	1.18

Domain topic importance		
1. Preparing researchers	4.48	0.90
2. Structural discrimination	4.52	0.91
3. Building on strengths in the community	4.61	0.84
4. Engagement methods	4.52	0.90

**Figure 1.**
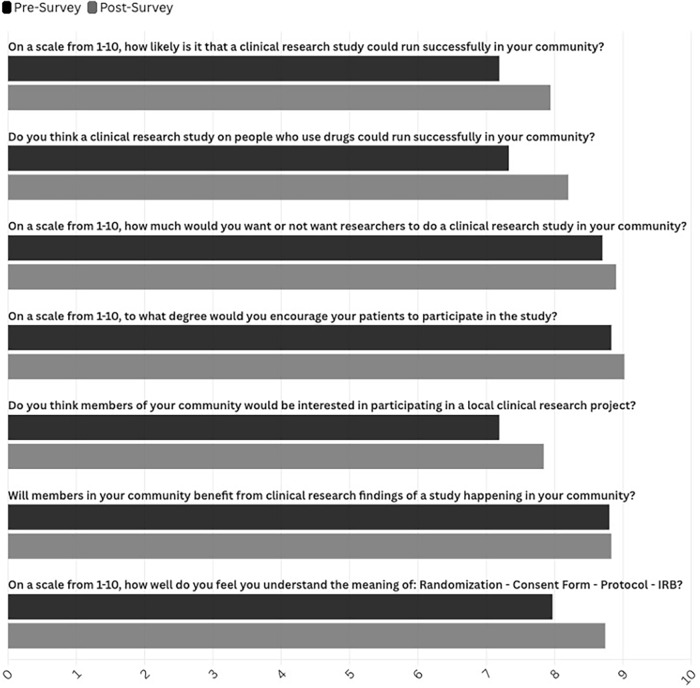
Attitudes and perceptions towards clinical research in the community: pre- and post-comparisons.

Pre- and post-survey comparisons are presented in Tables 1 and 6 and Figure 1. Wilcoxon signed-rank tests revealed no statistically significant differences across any of the seven items (all *p* > 0.05), with small to negligible effect sizes (*r* = 0.001–0.230). Scores improved directionally on most items, with the largest changes observed for perceived likelihood that clinical research with people who use drugs could run successfully in the community (pre: *M* = 7.27, SD = 2.22; post: *M* = 8.17, SD = 1.95) and perceived likelihood that clinical research could run successfully overall (pre: *M* = 7.20, SD = 2.39; post: *M* = 7.94, SD = 2.09). Three items showed minimal pre/post change, consistent with ceiling effects given high baseline scores: desire for researchers to conduct a study in the community (pre: *M* = 8.79), willingness to encourage patients to participate (pre: *M* = 8.88), and perceived community benefit from research findings (pre: *M* = 8.77). The Toolkit demonstrated adaptability across geographic regions and cultural backgrounds, with constructive results for CHWs serving both Hispanic communities in Southern New Mexico and American Indian communities in Northern New Mexico.

**Table 6.** Pre-/post-survey results: Wilcoxon signed-rank tests Table 6 long description.

Measure	*n*	Pre M (SD)	Post M (SD)	*W*	*Z*	*p*	*r*
Clinical research could run successfully in community	64	7.20 (2.39)	7.94 (2.09)	651.0	1.59	0.113	0.20
Clinical research with PWUD could run successfully	63	7.27 (2.22)	8.17 (1.95)	598.0	1.82	0.068	0.23
Desire for researchers to conduct study in community	62	8.79 (1.88)	8.89 (1.64)	469.5	0.30	0.768	0.04
Willingness to encourage patients to participate	64	8.88 (1.69)	9.02 (1.65)	466.0	0.58	0.562	0.07
Perceived community interest in local clinical research	64	7.28 (2.20)	7.84 (2.15)	633.5	1.35	0.179	0.17
Perceived community benefit from research findings	64	8.77 (1.61)	8.83 (1.55)	565.5	0.01	0.991	0.001
Research terminology understanding	54	8.22 (2.34)	8.72 (1.91)	243.0	1.18	0.239	0.16

### Qualitative results

Thematic analysis of open-ended responses from 78 total participants across both theater tests revealed three primary themes: toolkit effectiveness and usability, cultural appropriateness and community connection, and domain topic relevance.

#### Toolkit effectiveness and usability

Participants consistently emphasized the Toolkit’s practical utility, particularly noting Domain 1’s effectiveness in preparing researchers for community engagement. One participant noted, “This topic was explicit in its instructions, and it was clear to me what the researchers were explaining.” The small group format was valued, with participants appreciating “having facilitators in our group” to guide domain exploration. Participants offered important feedback for improvement, including suggestions to “use simpler language,” consider a “lower reading level,” and incorporate “graphics or pictures” to reduce written information. Time management emerged as a critical consideration, with participants emphasizing the need for “more time on the topics.”

#### Cultural appropriateness and community connection

Cultural competency emerged as fundamental to successful implementation. Participants stated that researchers must “know the culture that you are doing the study in” and “must be culturally competent and understand why community members may be hesitant to work with researchers.” Community involvement was identified as crucial, with distinct regional perspectives: Northern New Mexico participants emphasized elder involvement, while Southern New Mexico participants highlighted CHWs’ roles. Confidentiality concerns associated with rural communities were particularly salient, as one participant noted: “Making sure the community knows everything is confidential. Having worked with rural communities before, [many] are reluctant due to immigration status.”

#### Domain topic relevance

Theater testing yielded comprehensive feedback across all four domains. Domain 1 (Preparing Researchers) revealed important tensions between standardization and community-specific approaches. While some participants found the domain generalizable, others noted, “Minimal. Each community is unique,” underscoring that while basic engagement principles can be standardized, successful implementation requires flexibility and cultural competency.

Domain 2 (Structural Forms of Discrimination) generated robust discussion about systemic barriers. Participants noted its “very high impact” and emphasized that “to address it, you need to see the problem.” One participant observed, “The landscape of NM is constantly changing, and I am not always sure how to formulate that, but this was a strong process on non-discrimination.” Participants valued the domain’s emphasis on respect and non-judgment, noting how it “helps us remember the importance to not judge anyone.”

Domain 3 (Building on Strengths) was seen as “a door to increase services in the community.” However, some participants noted limitations: “It met the needs of some areas, but I struggled to see it come together for all communities here in NM.” The domain’s balance between acknowledging challenges and building on community assets was seen as crucial for meaningful engagement.

Domain 4 (Engagement and Communication Methods) received strong endorsement for practical utility. Participants noted that “all of the topics that involve the community help us” and that “communication is the important aspect to get the message out there for results.” One participant emphasized, “If you let the community know about the resources or help that is given, the community will respond.”

### Ripple effects mapping results

The REM process revealed three interconnected spheres of impact: shifts in researcher perspectives, enhancement of community–research relationships, and institutional and methodological transformation.

#### Shifts in researcher perspectives

Team members reported profound changes in their understanding of community engagement. As one researcher reflected, “What we thought would be a straightforward process of creating guidelines became a journey of understanding how communities view research differently.” The process highlighted how structural violence impacts individuals affected by SUD, revealing intersections not initially apparent to the research team. Researchers came to view community input not merely as informative but as fundamental to successful research design: “We had to unlearn our academic training to really hear what communities were telling us.”

#### Enhancement of community–research relationships

The REM process highlighted the fundamental importance of trust building and social relationships. Team members identified specific moments where their understanding of community dynamics shifted, particularly regarding informal networks and community gatekeepers. The mapping exercise illuminated the critical role of humility, with one team member observing, “We realized that our ‘expertise’ sometimes created barriers rather than bridges to community understanding.”

#### Institutional and methodological transformation

The REM process revealed how toolkit development transformed understanding of clinical trial implementation in community-based SUD research. Key insights included recognition that research benchmarks must be locally relevant rather than imported from different contexts; understanding that scale-appropriate research approaches may differ significantly from traditional clinical trial methods; appreciation for how regional differences affect implementation strategies; and recognition that success metrics may require redefinition in community contexts. Challenges around confidentiality – particularly in rural and border communities – catalyzed innovations including more flexible recruitment strategies respecting community rhythms and cultural practices and enhanced confidentiality protocols addressing specific community concerns.

## Discussion

The EXPLORE Toolkit development process revealed several key insights about the complexities of community engagement in SUD clinical trials research. What began as a targeted resource for researchers evolved into a versatile platform serving multiple stakeholders, from clinical researchers to community health workers and frontline responders. The multimethod evaluation approach, combining interviews, theater testing, and REM, yielded rich insights into both external engagement and internal team dynamics.

Theater testing proved particularly valuable for assessing complex behavioral health interventions, revealing critical insights about social determinants of health, trauma, and the importance of cultural sensitivity. Findings emphasize that successful community engagement requires deep understanding of local cultural contexts and power dynamics [4]. The critical role of community gatekeepers emerged as fundamental to research success, requiring careful consideration of power relationships, negotiation of boundaries, and collaborative knowledge construction, consistent with prior literature [31–33]. Cultural appropriateness proved essential for meaningful engagement; participants consistently emphasized the need for locally relevant approaches, including community testimonials and population-specific material adaptation [34]. The particular needs of tribal communities highlighted the importance of cultural protocols and indigenous knowledge systems [35]. Multiple language availability and attention to confidentiality concerns – especially in rural and border communities where immigration status may affect participation – emerged as critical success factors.

The EXPLORE Toolkit has potential to increase engagement of diverse populations in SUD clinical trials by helping community members understand what clinical trials entail. Trust building and community empowerment are critical to this process. The development process highlighted how successful research requires non-extractive, collaborative approaches and underscored the essential role of humility in community-engaged research. The toolkit’s effectiveness in empowering dialogue through education while maintaining accessibility addresses a critical need for resources that bridge the researcher–community divide. Theater testing across diverse New Mexico communities demonstrated the Toolkit’s adaptability, requiring effective cultural humility and respect [36].

The cross-institutional collaboration between UNM, NMSU, and NIH demonstrated the value of diverse perspectives in addressing complex health challenges. The REM process revealed how interdisciplinary dialogue enhanced understanding of SUD complexities in borderland communities, leading to innovations in presenting academic information accessibly and developing alternative methods for research interaction.

Perhaps most significantly, the REM process revealed a crucial, often overlooked aspect of community-engaged research: the transformation of researchers themselves. Team members documented how their own perspectives and approaches evolved, challenging traditional academic paradigms about expertise and knowledge construction. This willingness to adapt proved fundamental to the toolkit’s development and success. The REM process highlighted how meaningful community engagement requires researchers to embrace vulnerability, acknowledge their own biases, and remain open to fundamental shifts in their understanding of research practices [37]. This finding suggests that effective community-engaged research tools must not only guide community interaction but also support researcher growth and adaptation.

### Several limitations that warrant consideration

Testing was conducted in simulated rather than real-world settings, potentially limiting understanding of implementation challenges in practice. The geographic focus on the Southwestern United States may limit generalizability to other regions. While the toolkit was tested with particular American Indian and Spanish-speaking populations in New Mexico, there is significant heterogeneity within these groups both in the Southwestern United States and throughout the country. This diversity means there are other strengths, risk factors, and culturally specific values and insights that should be considered beyond what is presented here. Initial testing focused primarily on CHWs rather than researchers, and the long-term impact and sustainability of the toolkit require further evaluation. Finally, the toolkit’s effectiveness across different SUD contexts needs further exploration.

### Future directions

This paper marks the beginning rather than the conclusion of the EXPLORE Toolkit’s development. Future priorities include real-world implementation studies across diverse urban and rural settings and cultural contexts beyond the Southwest and systematic evaluation frameworks measuring both immediate and long-term impact on research participation and community engagement. A dissemination plan is underway leveraging NIDA CTN, social media, and other platforms. Ongoing toolkit refinements based on user experience evaluation include replacing the term “domain” for clarity, reformatting reflection sheets, and adding QR codes to facilitate sharing. Formal feedback mechanisms, protocols for regular content updates, and community review boards to guide adaptations will help ensure the toolkit remains a living document. Online training for different partner groups and digital platforms for user networking may further support effective implementation.

### Conclusion

While the EXPLORE Toolkit does not claim to be a comprehensive solution to community engagement challenges in clinical trials research, it represents a significant step toward bridging researcher–community gaps in SUD research. By facilitating meaningful dialogue and promoting community agency in healthcare research, the Toolkit contributes to the broader goal of ensuring more inclusive and effective SUD research. Its development demonstrates the value of interdisciplinary collaboration and community-centered approaches in addressing complex public health challenges. The toolkit is freely available at https://www.exploretoolkit.org/.

## Data Availability

The datasets generated and/or analyzed during the current study are available in the Figshare repository, https://doi.org/10.6084/m9.figshare.29452202.v1.

## Table 1. Long description

The table compares various toolkits used for public health, community engagement, and patient-centered outcomes. It has five columns: Name of toolkit, Mission/aim of toolkit, Methods/principles of engagement, Components of toolkit, Other useful info, and Unique elements. The table contains 17 rows, each representing a different toolkit. Row 1: ReachNet, The involvement of patients, caregivers, clinicians, and other healthcare stakeholders throughout the research process from topic selection through design and conduct of research, to dissemination of results. Helping people make informed decisions throughout the healthcare delivery and health outcomes, Patients can contribute to and prioritize research topics; patients can depend on being subjects and more as consultants; be connected to the clinical research teams in meaningful ways, Script for medical assistant, Data use policy, Patient Consent Forms, This is an upstream assistant. Key is: who controls the narrative. How to include voices input for trials that are already designed, Clinic champion (pg. 18); a role to integrate patients, providers and other stakeholders. Row 2: Brandeis Community Engagement Studio (CES) toolkit, Providing an outline for an interactive, consultative model for community involvement in which community experts are seen as collaborators in research design, not subjects of studies, Key members of CES team: Community navigator (navigates community relationships with academic group); science navigator (works with investigators to facilitate communication with stakeholders); manager (works with partners to ensure effective implementation); Facilitator for Studio testing, Researcher presenting research, and community experts (pgs. 12-18 for more details), Facilitator CES guide; Tools for Recruiting Researcher CES guide; Tools for recruiting community experts; Community Expert CES guide; Consent forms; CES evaluation survey, Good table on page 7 distinguishing between focus group and CES. Row 3: Gathering of Native Americans (GONA) toolkit, Complementing the guide above, this toolkit offers dozens of materials for team building, trust building, and engagement materials for facilitators working with Native American groups, Toolkits for Belonging, Mastery, Interdependence, Generosity and Healing from Organizational Healing; also one geared towards youth, Offers concrete examples of what centering Indigenous ways of knowing looks like in practice. Row 4: Rain barrel communications participatory research toolkit, A toolkit with dozens of specific tools for participatory action research, with outlines of the tools, reflections of using the tools, and the insights, and the insights. It is used for myriad health issues across the world, including female genital mutilation, nutrition, AIDS, malaria, etc., They can be used to conduct participatory situation assessments; monitor the extent to which interventions are being implemented according to plan or to meet their effectiveness, i.e., change behaviors and social outcomes. The strength of these tools lies in the fact that they can be integrated directly into individual and social change intervention programming, providing participants the ability and skills to create and analyze data. Many of the tools are specifically designed to build teamwork and make research an enjoyable exercise. Additionally, these tools can be used to examine societal and cultural factors which are difficult to understand and decipher using traditional methods. This is both for participants and researchers to see the world in a different light. (direct citation from intro to document), The toolkit is a list of all of the tools used. Description of tool, rationale, application, and tips for use. See document for all of the tools, Too many tools to look at, could be useful for teams to go through and see if any are useful. This is a good example of how to lay out tools, how to apply, and how to use and what to expect.

Navigate back to Table 1..

## Table 2. Long description

A table with five rows and two columns. The first column lists domain names, and the second column provides descriptions. Row 1: Domain 1: preparing researchers to go into communities, Introduces the toolkit’s purpose. Addresses three primary user groups: experienced researchers seeking greater participant diversity; clinicians and professionals involved in clinical trial recruitment or referral; and community health workers (CHWs) who interface directly with community members. Row 2: Domain 2: structural factors that influence participation, Presents five interconnected thematic areas – geography; racism, skepticism, and distrust of researchers; economic and material factors; education, stigma, and health literacy; and gender and sexuality – illustrating how social/economic policies, healthcare resource distribution, and social stratification create barriers to participation among marginalized populations. Row 3: Domain 3: building on strengths to support participation, Operates in parallel with Domain 2. Offers a framework for reframing perceived barriers as community assets and provides practical strategies for leveraging these strengths in clinical trial design and implementation. Row 4: Domain 4: engagement and communication methods, Features two illustrative vignettes depicting hypothetical researcher-community interactions, highlighting the critical role of language, context, and social dynamics in community engagement. Row 5: Domain 5: tools and resources, Provides comprehensive implementation resources: tables of tools organized by domain and theme, links to existing toolkits, original team-developed tools, and curated external resources supporting focus group facilitation, trust building, clinical trial education, and pre/post-trial evaluation.

Navigate back to Table 2..

## Table 3. Long description

A table with three columns and five rows, detailing themes and subthemes related to structural forms of discrimination. The columns are labeled Theme, Subtheme, and Example. Row 1: Theme Geography, Subtheme Transportation instability, Example Having limited access to transportation could make it challenging for an individual to make it to their appointments for clinical trials if they are conducted in person. Row 2: Theme Racism, skepticism, and distrust of researchers, Subtheme Skepticism of the medical system, Example Medical providers often operate under outdated approaches with stigmatizing beliefs about substance use and substance use treatment which cause patients to feel judged and avoid healthcare systems. Row 3: Theme Economic/material factors, Subtheme Poverty, Example Not having enough money to live comfortably could impact an individual’s decision to participate in clinical research due to time constraints and perceived value for giving their time based on the compensation. Row 4: Theme Education, stigma, and health literacy, Subtheme Health misinformation/literacy, Example Misinformation has been a growing concern for the scientific community with the widespread use of social media and news platforms, which are pervasive and often provide incorrect information. This prevalence could limit the trust that an individual has in the motivation for the trial and potential outcomes. Row 5: Theme Gender and sexuality, Subtheme Gender Identity, Example Historically, female/woman-identifying individuals absorb much of the household labor and responsibility within a family unit, including childcare, which can limit the amount of time they are able to give to participate in clinical trials.

Navigate back to Table 3..

## Table 4. Long description

A table with three columns and three rows. The columns are labeled Barrier, Strength, and Strategies. The rows provide detailed information under each column header. Row 1: Barrier, In smaller communities it is more likely that people know each other or of each other. This can discourage participation in substance use research to avoid stigma and discrimination. Strength, Video conferencing and other virtual communication resources have become more common in the wake of the COVID-19 pandemic. These tools facilitate more discrete participation in research. Strategies, Virtual resources can reduce potential stigma against participating in clinical trial research as well as reduce barriers such as geographic distance from a research site. Virtual technology allows participants to be involved with privacy. Trials may be designed to reduce the amount of fact-to-face contact. There are a growing number of resources available for Decentralized Clinical Trials that may be well suited for rural areas. If your research takes place at a fixed site, consider how you can create a private entrance, or backdoor into the site to maximize privacy and confidentiality.

Navigate back to Table 4..

## Table 5. Long description

A table titled ‘Toolkit Impressions and importance of toolkit domain topics’ with two columns: Score with sub-columns Mean and SD, and Impressions and views with sub-columns Toolkit usability, Cultural relevance, Promotes dialogue, and Domain topic importance. The table has six rows and two columns. Row 1: Toolkit usability, Mean: 4.26, SD: 0.89. Row 2: Cultural relevance, Mean: 4.07, SD: 1.11. Row 3: Promotes dialogue, Mean: 4.10, SD: 1.18. Row 4: Domain topic importance, Mean: N/A, SD: N/A. Row 5: 1: Preparing researchers, Mean: 4.48, SD: 0.90. Row 6: 2: Structural discrimination, Mean: 4.52, SD: 0.91. Row 7: 3: Building on strengths in the community, Mean: 4.61, SD: 0.84. Row 8: 4: Engagement methods, Mean: 4.52, SD: 0.90.

Navigate back to Table 5..

## Table 6. Long description

A table comparing pre- and post-survey measures related to clinical research in the community. The table has 8 rows and 7 columns. The columns are labeled Measure, n, Pre M (SD), Post M (SD), W, Z, p, and r. The rows are labeled with different measures related to clinical research. Row 1: Clinical research could run successfully in community, n: 64, Pre M: 7.20 (SD: 2.39), Post M: 7.94 (SD: 2.09), W: 651.0, Z: 1.59, p: 0.113, r: 0.20. Row 2: Clinical research with PWUD could run successfully, n: 63, Pre M: 7.27 (SD: 2.22), Post M: 8.17 (SD: 1.95), W: 598.0, Z: 1.82, p: 0.068, r: 0.23. Row 3: Desire for researchers to conduct study in community, n: 62, Pre M: 8.79 (SD: 1.88), Post M: 8.89 (SD: 1.64), W: 469.5, Z: 0.30, p: 0.768, r: 0.04. Row 4: Willingness to encourage patients to participate, n: 64, Pre M: 8.88 (SD: 1.69), Post M: 9.02 (SD: 1.65), W: 466.0, Z: 0.58, p: 0.562, r: 0.07. Row 5: Perceived community interest in local clinical research, n: 64, Pre M: 7.28 (SD: 2.20), Post M: 7.84 (SD: 2.15), W: 633.5, Z: 1.35, p: 0.179, r: 0.17. Row 6: Perceived community benefit from research findings, n: 64, Pre M: 8.77 (SD: 1.61), Post M: 8.83 (SD: 1.55), W: 565.5, Z: 0.01, p: 0.991, r: 0.001. Row 7: Research terminology understanding, n: 54, Pre M: 8.22 (SD: 2.34), Post M: 8.72 (SD: 1.91), W: 243.0, Z: 1.18, p: 0.239, r: 0.16.

Navigate back to Table 6..
